# New Therapeutic Strategy and Innovative Lubricating Ophthalmic Solution in Minimizing Dry Eye Disease Associated with Cataract Surgery: A Randomized, Prospective Study

**DOI:** 10.1007/s12325-020-01288-z

**Published:** 2020-03-17

**Authors:** Paolo Fogagnolo, Eleonora Favuzza, Daniele Marchina, Michela Cennamo, Roberto Vignapiano, Chiara Quisisana, Luca Rossetti, Rita Mencucci

**Affiliations:** 1 Eye Clinic, ASST Santi Paolo Carlo, San Paolo Hospital, Milan, Italy; 2 Department of Health Sciences, University of Milan, Milan, Italy; 3 Department of Oto-Neuro-Ophthalmological Surgical Sciences Eye Clinic, University of Florence, Florence, Italy

**Keywords:** Cataract surgery, Dry eye disease, Lubricating eye drops, Ocular surface, Ophthalmology

## Abstract

**Introduction:**

To evaluate the effects of a new lubricating, antioxidant solution (VisuEvo^®^) on dry eye disease (DED) in patients undergoing cataract surgery.

**Methods:**

Patients requiring cataract surgery with either healthy ocular surface or mild DED (tear break-up time, TBUT > 7, Schirmer *I* test > 15 mm/5 min) were enrolled in this multicenter, open-label, randomized, prospective study. Scheduled visits were 2 weeks before surgery (screening), day of surgery (V0), week 1 (V1), and 2 (V2) after surgery. VisuEvo^®^ was self-administered three times daily for the whole study duration (group A); the control group (group B) had no tear substitute administration. The primary endpoint was the change in TBUT over time; the secondary endpoints were changes in Ocular Surface Disease Index (OSDI), ocular surface staining, the Schirmer *I* test, and osmometry.

**Results:**

A total of 45 patients were included (group A, 23; group B, 22; age 74 ± 8 years). At the screening, TBUT was similar between the groups (group A, 8.5 ± 1.8 s; group B, 7.8 ± 0.7, *p *= 0.11). At the scheduled visits, TBUT increase vs screening visit was significantly higher in group A: +1.2 s at V0, +1.4 s at V1, and +1.9 s at V2 (*p *< 0.01). Also, OSDI was significantly lower in group A at V0, V1, and V2 (*p *< 0.027). After surgery, corneal staining was absent in 65–78% of group A compared with 54–59% in group B. The two groups did not show any significant differences of osmometry and the Schirmer I test.

**Conclusions:**

The ocular surface was more protected and quickly restored from surgery when VisuEvo^®^ was used from 2 weeks preoperatively to 2 weeks postoperatively.

**Trial registration:**

ClinicalTrials.gov identifier, NCT03833908

## Key Summary Points

**Table:** 

**Why carry out this study?**
An adequate ocular surface preparation is needed in patients scheduled for cataract surgery, both in individuals with ocular surface disease and a healthy ocular surface. This practice is poorly adopted.
Cataract surgery often induces or exacerbates DED, especially in the elderly.
**What was learned from the study?**
The use of a prophylactic lubricating treatment 2 weeks before scheduled cataract surgery, and continued 2 weeks postoperatively, protected patients from iatrogenic DED.
The preoperative administration of the new ophthalmic solution, VisuEvo^®^, guaranteed optimal ocular surface health until the day of surgery and preserved patients from the natural course of DED postoperatively.

## Introduction

Cataract is a leading cause of visual impairment in all regions of the world [1], and surgery is the only treatment choice for visually disabling cataracts [2]. Regardless of the surgical technique employed (e.g., phacoemulsification, manual small-incision cataract surgery, or extracapsular cataract extraction), cataract surgery has been shown to induce or exacerbate dry eye disease (DED) [3]. In most cases, DED is transient after surgery. Dry eye symptoms may occur at variable periods after uncomplicated phacoemulsification, combined with a decrease in tear break-up time (TBUT) and an increase in ocular surface staining [4–6]. Also, cataract surgery may trigger an entry in a vicious cycle of chronic DED, which occurs with a prevalence of about 10% [5].

The pathophysiological mechanisms underlying cataract surgery-induced DED are multifactorial and include the use of preoperative prophylactic medications, topical anesthetics and antiseptics, exposure desiccation, possible light toxicity from the operating microscope, corneal nerve transection, increase of inflammatory factors, goblet cell loss, and meibomian gland dysfunction [6–9]. The surgical trauma related to cataract surgery is associated with the production of oxygen-free radicals, proteolytic enzymes, prostaglandins, leukotrienes and inflammatory cytokines, which may affect corneal sensitivity, increase inflammation and contribute to tear film instability [7]. Of note, conjunctival goblet cell density decreased remarkably following uncomplicated cataract surgery and did not return to baseline even 3 months postoperatively [3].

A healthy ocular surface is crucial to achieving the best outcome in cataract surgery. Ocular surface preparation is crucial in patients with established ocular surface disease, and it is also helpful in those with minimal signs or symptoms of the surface disease. As the incidence and severity of DED may increase after cataract surgery [4], an assertive approach in the management of ocular surface disease since the pre-operative phases is recommendable in the majority of patients. However, there is a discrepancy between the high number of commercially available lubricating eyedrops and the low number of studies exploring their clinical usefulness. Only scarce evidence is currently available on the efficacy of these types of eye drops in reducing DED after cataract surgery. This study reports on the potential clinical benefits of a new approach to cataract surgery-associated DED based on the administration of an innovative preservative-free, antioxidant activity ophthalmic solution (VisuEvo^®^) pre-operatively and some weeks after surgery.

## Methods

This was a multicenter, pre-marketing, open-label, randomized, prospective study. From November 29, 2018 to June 17, 2019, consecutive patients scheduled to receive cataract surgery with normal ocular surface or mild DED (including subclinical DED) were enrolled at the Eye Clinic of Careggi Hospital, University of Florence, Italy, and at San Paolo Hospital—SST Santi Paolo e Carlo of Milan, University of Milan, Italy. Informed consent was obtained from all participants, and the study was reviewed and approved by the Ethics Committees of two Institutions (Comitato Etico Area Vasta Centro, Comitato Etico Milano Area 1). The study was conducted in accordance with this approval and national regulations, and adhered to the tenets of the Declaration of Helsinki as revised in 2013. The trial was registered at https://www.clinicaltrial.gov (identifier: NCT03833908).

The study design included four visits: 2 weeks before the planned cataract surgery (screening visit), day of cataract surgery (baseline visit), and then at weeks 1 and 2 after surgery (post-surgery visits 1 and 2, respectively). Patients were enrolled in the study at the screening visit, and randomized with a 1:1 ratio (by means of a list of random numbers) to one of two groups each of 23 patients: group A (investigational group), treated with VisuEvo^®^ administered three times daily starting 2 weeks before surgery until 2 weeks post-surgery for a total of 4 weeks; and group B (comparator group), without any tear substitute administration.

Three days before surgery, the patients of both groups received standard treatment with ofloxacin. Soon after surgery, they had a standard postoperative treatment (topical dexamethasone for 10 days associated with ofloxacin for 7 days). Patients were allowed to continue any systemic or local medications for their concomitant diseases. Before the screening visit for all patients, no topical ophthalmic medication, including lubricating eye drops, were administered for at least 4 days. Major inclusion criteria encompassed adults of both genders diagnosed with cataract requiring surgery, with healthy ocular surface or mild DED (including subclinical DED) with TBUT > 7, and Schirmer test > 15 mm/5 min. Subjects were excluded from enrollment if they carried neuropathic DED (e.g., diabetes, long-standing contact lens wearing, previous ocular herpes infections, previous eye surgery), proven or suspected glaucoma or ocular hypertension, Sjögren syndrome and/or other autoimmune diseases, complicated cataract, corneal diseases, surface eyes disturbances (e.g., past or active cicatricial conjunctivitis, ocular surface burns, corneal trauma), keratinization of the eyelid margin and/or other functional and anatomic eyelid abnormalities.

The primary objective of the study was to compare the change over time in TBUT between the two treatment groups. The secondary objectives were the performance of VisuEvo^®^ in reducing changes in ocular surface staining (according to a modified Oxford Scale considering also the staining of the surgical incisions) [10], eye disability determined by the Ocular Surface Disease Index questionnaire (OSDI; ©1995, Allergan, Irvine, CA, US) [11], Schirmer I test, and osmometry. The equipment used for assessing the clinical variables was the slit lamp ophthalmoscopy, the tear film osmometer, the fluorescein strips, and the Schirmer test strips without anesthesia.

The safety profile was assessed by monitoring the occurrence of adverse events (AEs).

Patients were asked to self-administer study medications during the study. VisuEvo^®^ is an ophthalmic solution with antioxidant activity (scavenger of oxygen free radicals) that uses an innovative liposomal nanodispersion associated with vegetable oil rich in omega 3 (docosahexaenoic acid and eicosapentaenoic acid), vitamin D and vitamin A palmitate. VisuEvo^®^ is a 10-ml, preservative-free, multidose bottle provided with a dispenser capable of delivering the drops in sterile conditions and preserving the content from exogenous contamination during use (Novelia^®^ System). The structure of the ophthalmic solution is thought to be effective in most forms of DED apart from Sjögren disease, with a significant hyperevaporative component.

All patients received cataract surgery according to standard operating procedures: pupil dilation with three drops of Visumidriatic Tropicamide^®^ (Visufarma, Italy) and Visumidriatic Phenilephrine^®^ (Visufarma), preparations with three drops of single-dose oxybuprocaine (Novesina^®^ 4 mg/ml; Laboratoires Thea, France), 10% iodine solution for 15 min on the eyelid skin and 5% iodine solution (Oftasteril^®^; Alfa Intes, Italy) for 5 min on the conjunctival sac. Standard phacoemulsification with clear cornea temporal incision was performed.

In this study, no intraoperative and postoperative complications occurred.

A sample size of 42 patients was estimated to provide 80% power to detect a significant difference between treatment arms by a two-sided *t* test for unpaired data (type I error set to 0.05). A 2-s change in TBUT has been considered as clinically relevant and, based on previous studies [13–15], 2.2 s has been assumed as the standard deviation. Planning to randomize a total of 46 patients (23 in each treatment arm) allowed for a 5% drop-out rate (sample size estimation was performed using SAS software v.9.4). In the study analysis, the following populations were considered: Intent-to-Treat (ITT) set, consisting of all randomized patients; and Safety Analysis (SA) set, consisting of all randomized patients, according to their actual treatment.

## Results

Forty-six subjects were enrolled; 45 patients completed the study, 23 in group A and 22 in group B. One subject withdrew their informed consent between screening and the baseline visit. This patient was excluded from the analysis because they did not provide post-baseline data. The two groups were well matched for gender, ethnicity, age, height, weight, and the time elapsed from cataract diagnosis, and no statistically significant differences were observed. The demographic results are described in Table 1.

**Table 1:** Demographic results (ITT population)

	Group A (*n* = 23)	Group B (*n* = 22)	Total (*n* = 45)
Gender *n* (%)			
Male	7 (30.4%)	8 (36.4%)	15 (33.3%)
Female	16 (69.6%)	14 (63.6%)	30 (66.7%)
Ethnic group			
Caucasian	23	22	45
Age (years)			
Mean ± SD	73 ± 7	76 ± 8	74 ± 8
Median (range)	72 (52–88)	77 (54–86)	76 (52–88)
Height (cm)			
Mean ± SD	163 ± 10	163 ± 8	163 ± 9
Median (range)	164 (146–180)	160 (140–175)	162 (140–180)
Weight (kg)			
Mean ± SD	69 ± 16	71 ± 13	70 ± 14
Median (range)	68 (45–95)	68 (45–100)	68 (45–100)
Time elapsed from cataract diagnosis (days)^a^			
Mean ± SD	600 ± 291	763 ± 321	679 ± 314
Median	732	765	740

^a^Time elapsed from cataract diagnosis was calculated as (Cataract diagnosis date—Visit − 1 date) + 1

**Fig. 1:**
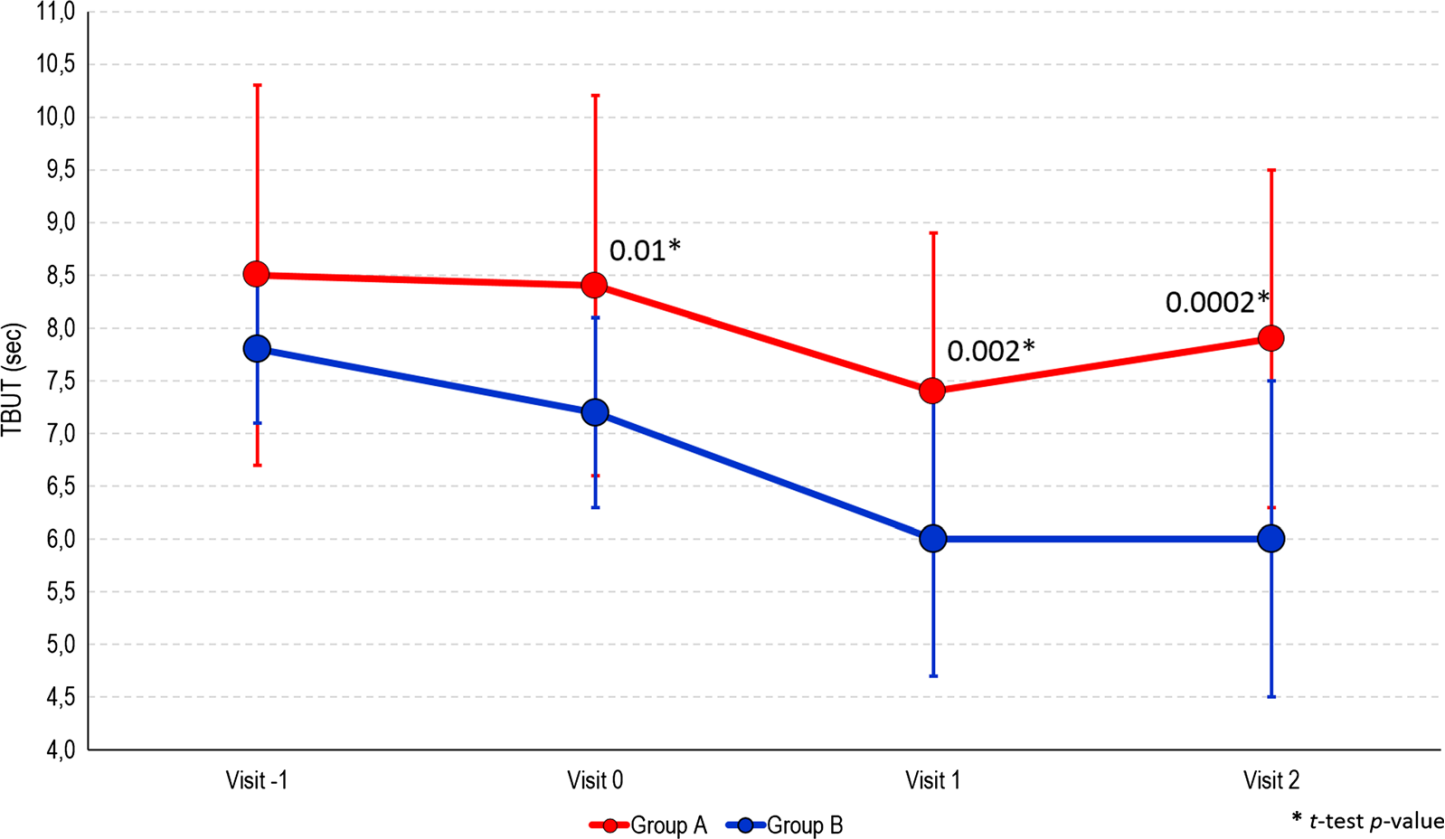
Trend of TBUT (s) among study visits by study treatment group

The results of the study are summarized in Table 2. At the screening visit, the TBUT values were similar in group A and in group B (8.5 ± 1.8 and 7.8 ± 0.7 s, respectively, *p *= 0.11). At baseline before the cataract surgery (visit 0), the mean TBUT value in group A was comparable to the one at the screening visit, while a decrease of 0.6 s was observed in group B. The difference between the two groups was statistically significant (*p *= 0.01) (Fig. 1). One week after surgery (visit 1), TBUT decreased in the group A (7.4 ± 1.5 s) compared to previous visits, whereas it decreased in the group B to 6.0 ± 1.3 s (*p *= 0.002). At visit 1, TBUT values were worse than at the screening visit in 57% of patients in group A and 91% in group B. Two weeks after surgery (visit 2), TBUT showed a trend toward the initial mean value observed at the screening visit in group A, while it further decreased in group B (*p *= 0.0002). The difference between the two groups further increased compared to the visit 1 (Fig. 1). Comparing the beginning and the end of the study, TBUT worsened in 57% of patients in group A, and 82% in group B (Table 3). Stratifying the patients into four classes according to the severity of TBUT (e.g., < 5 s, 5–7 s, 8–9 s, ≥ 10 s), at the screening, there were similar distributions in the two groups. However, during the following visits, a progressive advantage of group A over group B emerged (Table 4).

**Table 2:** Primary and secondary efficacy variables at each study visit (ITT population)

Variables at study visits	Group A (*n* = 23)	Group B (*n* = 22)	*p* value
TBUT—mean ± SD			
Visit − 1	8.5 ± 1.8	7.8 ± 0.7	0.11
Visit 0	8.4 ± 1.8	7.2 ± 0.9	0.01
Visit 1	7.4 ± 1.5	6.0 ± 1.3	0.002
Visit 2	7.9 ± 1.6	6.0 ± 1.5	0.0002
OSDI Questionnaire Score—mean ± SD			
Visit − 1	13 ± 8	14 ± 8	0.55
Visit 0	6 ± 5	11 ± 7	0.01
Visit 1	8 ± 7	16 ± 12	0.014
Visit 2	9 ± 8	16 ± 11	0.027
Osmolimetry test—mean ± SD			
Visit − 1	305 ± 17	303 ± 13	0.77
Visit 0	306 ± 17	299 ± 17	0.21
Visit 1	302 ± 18	304 ± 17	0.79
Visit 2	304 ± 16	302 ± 13	0.66
Schirmer *I* test—mean ± SD			
Visit − 1	20 ± 6	18 ± 5	0.31
Visit 0	20 ± 7	16 ± 5	0.08
Visit 1	16 ± 6	16 ± 8	0.84
Visit 2	18 ± 8	16 ± 7	0.52
Staining grade with fluorescein—*n* (%)			
Visit 1			
0	18 (78.3%)	13 (59.1%)	
1	4 (17.4%)	5 (22.7%)	
2	1 (4.3%)	4 (18.2%)	
Visit 2			
0	15 (65.2%)	12 (54.5%)	
1	7 (30.4%)	5 (22.7%)	
2	1 (4.3%)	5 (22.7%)	

*Visit − 1* screening;* Visit 0 * baseline;* Visit 1 * week 1;* Visit 2 * week 2

**Table 3:** Number and proportion of patients improved, stablilized and worsened in TBUT values during study visits compared to screening visit (visit − 1) by treatment group (ITT population)

	Group A *n* (%)			Group B *n* (%)		
	Improved	Stabilized	Worsened	Improved	Stabilized	Worsened
Visit − 1 vs Visit 0	5 (22)	8 (35)	10 (43)	3 (14)	10 (45)	9 (41)
Visit − 1 vs Visit 1	2 (9)	8 (35)	13 (57)	0 (0)	2 (9)	20 (91)
Visit − 1 vs Visit 2	6 (26)	4 (17)	13 (57)	1 (5)	3 (14)	18 (82)

*Improved* difference between two TBUT values > 0; *Stabilized*  difference between two TBUT values = 0; *Worsened*  difference between two TBUT values < 0

**Table 4:** Stratification of patients of both treatment groups into classes of TBUT severity

TBUT classes	Visit -1			Visit 0			Visit 1			Visit 2		
	Group A (*n* = 23) *n* (%)	Group B (*n* = 22) *n* (%)	*p*^a^	Group A (*n* = 23) *n* (%)	Group B (*n* = 22) *n* (%)	*p*^a^	Group A (*n* = 23) *n* (%)	Group B (*n* = 22) *n* (%)	*p*^a^	Group A (*n* = 23) *n* (%)	Group B (*n* = 22) *n* (%)	*p*^a^
< 5 s	0 (0.00)	0 (0.00)	0.17	0 (0.00)	0 (0.00)	0.02	0 (0.00)	4 (18.2)	0.01	0 (0.00)	3 (13.6)	0.004
5–7 s	6 (26.1)	7 (31.8)		8 (34.8)	14 (63.6)		12 (52.2)	15 (68.2)		11 (47.8)	17 (77.3)	
8–9 s	13 (56.5)	15 (68.2)		9 (39.1)	8 (36.4)		10 (43.5)	3 (13.6)		7 (30.4)	2 (9.1)	
≥ 10 s	4 (17.4)	0 (0.00)		6 (26.1)	0 (0.00)		1 (4.3)	0 (0.00)		5 (21.8)	0 (0.00)	

^a^Fisher *p* value

**Fig. 2:**
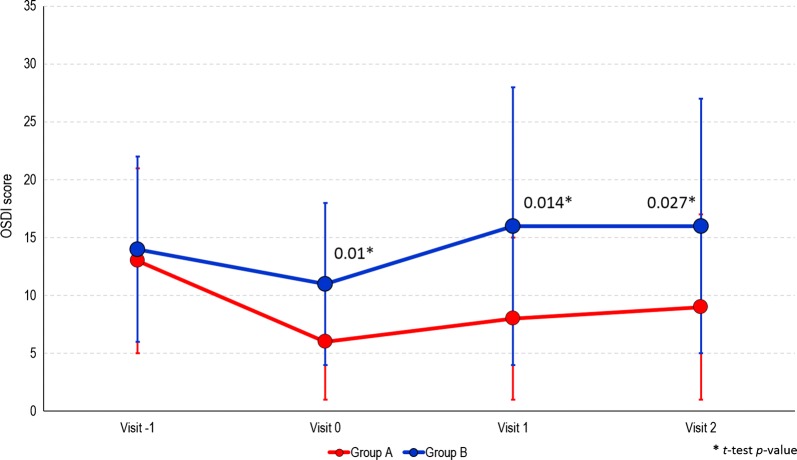
Trend of OSDI Questionnaire Score among study visits by study treatment group

At the screening visit, OSDI scores were similar in both groups. OSDI overall decreased the day of the surgery; the reduction was significantly higher in group A compared with group B (*p *= 0.01). During the two post-surgery visits, a small increase of OSDI score was found in group A;, while the increase was greater in the unprotected group B (Fig. 2). The differences in mean OSDI scores between the groups were statistically significant at both visits 1 and 2 (*p *= 0.01 and *p *= 0.027, respectively).

**Fig. 3:**
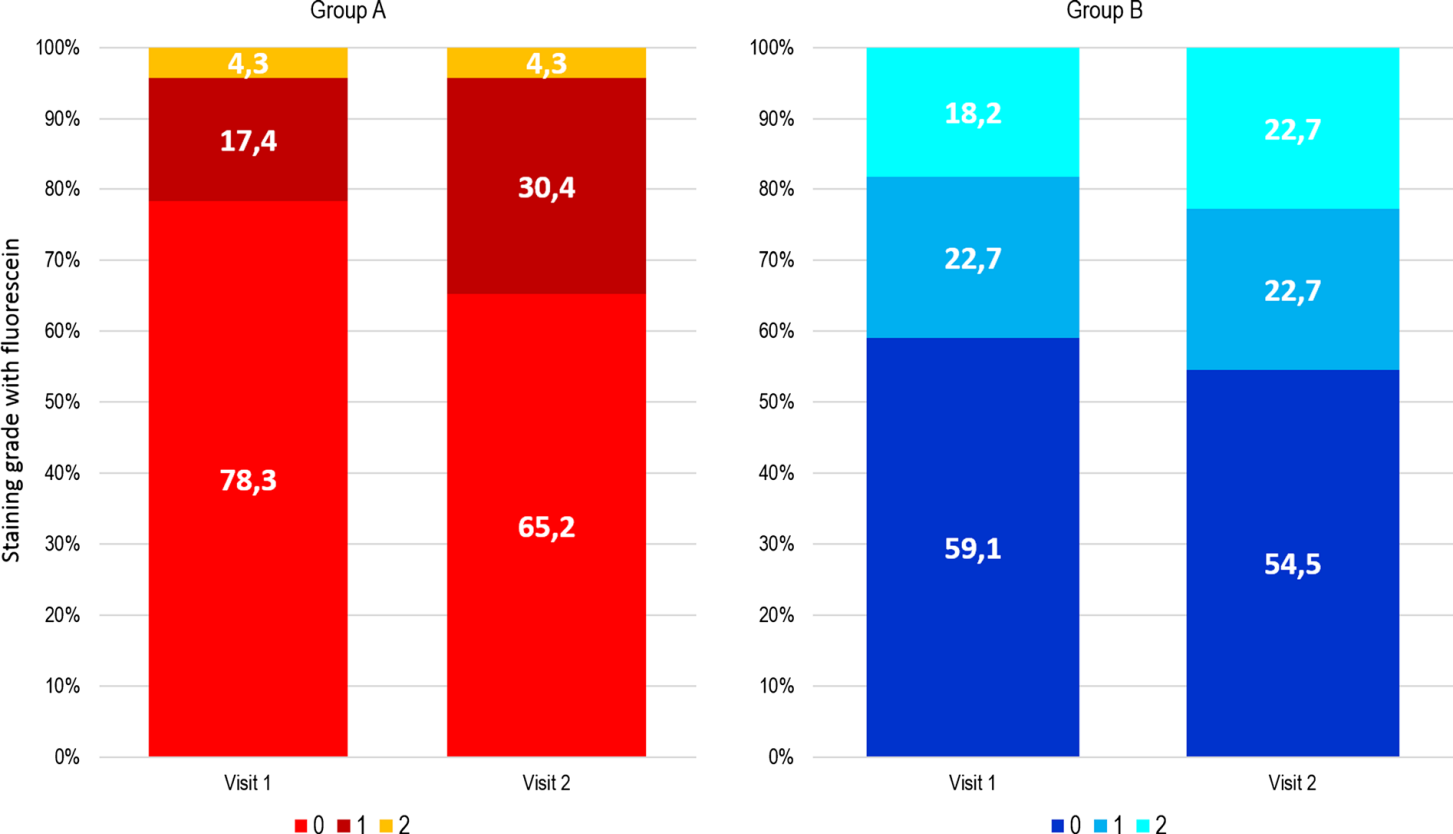
Distribution of patients assessed by staining grade with fluorescein by study group at Visit 1 and Visit 2 post-surgery

After surgery, fluorescein staining showed a much higher proportion of patients with optimal ocular surface protection in the group treated with VisuEvo^®^ (group A) compared to group B (Table 2). A greater percentage of patients (78 and 65% at visits 1 and 2, respectively) had grade 0 in group A versus those in group B (59 and 54%, respectively). Also, a minor proportion of patients had a staining grade 2 in group A at both visits (4.3%) versus those in group B (18 and 23%, respectively) (Fig. 3).

Osmometry was similar in the two groups at screening, and no statistically significant difference was observed between the groups at any visit (Table 2). Also, the mean values of the Schirmer *I* test were similar between groups throughout the study, and no statistical significance between groups at any visit was detected (Table 2).

During the study, three patients (6.7%) in group A reported four ocular AEs: iris incarceration in the corneal wound, iridocyclitis, increased eye pressure after surgery, and conjunctivitis. No AE met the criteria for being classified as severe or serious. Only the patient experiencing increased eye pressure after surgery reported the AE to be of moderate intensity.

## Discussion

Two of the most relevant risk factors for DED are anterior segment surgery [3, 16, 17] and increased age [3]. As cataract surgery is commonly performed on the elderly, DED frequently occurs after this procedure. Approximately one-third of individuals experience mild or greater DED symptoms after surgery [18]. Of note, the DED prevalence may be different depending on the diagnostic criteria. A recent report by DEWS recommends non-invasive diagnostic tests [19]. Nevertheless, TBUT, together with the OSDI scores and Schirmer I test, is still the most commonly used parameter to ascertain the presence of DED and monitor its changes over time [20, 21]. Our study population undergoing cataract surgery had similar features to that of previous studies [20, 21]. Of the whole study population, individuals with normal ocular surfaces (TBUT > 7 s) and no relevant DED symptoms (OSDI ≤ 12) were just 38% at baseline.

In our study, patients in the comparator group showed the natural course of DED after surgery. These patients had a detriment of TBUT at the moment of surgery, possibly due to pre-surgery preparation with antibiotics, which are known to be toxic to the ocular surface [22, 23]. In these patients, cataract surgery was associated with further worsening of the ocular surface health (TBUT, staining), and the effect was clear after 1 week, without signs of improvement at the 2-week visit.

Conversely, patients receiving the ophthalmic solution VisuEvo^®^ were more protected from iatrogenic DED. This ophthalmic solution was able to fully counteract the toxic effects of eye drops used to prepare patients for surgery, so that they received surgery with overall normal homeostasis of the ocular surface (as TBUT was similar to baseline). Surgery also worsened the ocular surface on these patients, but the effect was significantly lower than in group B (Fig. 1). The TBUT differences between the two groups progressively increased during the study up to the last visit, when it was the largest. TBUT findings were parallel to the trend of OSDI score, showing a greater reduction of ocular disability compared to patients unprotected with the ophthalmic solution. The use of VisuEvo^®^ was also associated with a larger percentage of subjects with no corneal staining at fluorescein after surgery (65–78% in group A vs. 54–59% in group B; Fig. 3).

The results of the current study are consistent with previous reports, which have shown that cataract surgery can lead to worsened all dry eye test values regardless of a previous DED [3]. To assess the efficacy of the new ophthalmic solution in protecting against the ocular surface damages, we used conventional dry eye tests. In our study, the TBUT, OSDI score and Oxford ocular surface staining system agreed in estimating DED occurrence and severity [5], while Schirmer I and osmometry did not contribute in evaluating our study population. The low performance of Schirmer I was expected given the normal function of the main lacrimal gland at baseline. Also, osmometry did not show any statistical difference in the two groups during the study, with negligible changes after surgery and in the group treated with VisuEvo^®^. The limited relevance of osmometry in this study may be the consequence of several factors. On the basis of inclusion and exclusion criteria, the study population had no inflammatory conditions affecting the ocular surface before surgery; also, a high percentage of subjects had no DED before the study (see mean TBUT at visit − 1, Table 1). For these reasons, mean osmometry at visit − 1 was normal as expected, which made a further amelioration of the test unlikely. Moreover, the postoperative use of steroids may have ameliorated the conditions of the ocular surface, thus stabilizing osmometry, both mitigating the negative effects of surgery and, in the treated group, the positive effects of VisuEvo^®^.

Many studies have shown the effects of various tear film substitutes [12, 24–26, 28–30] and cyclosporine [27] in ameliorating iatrogenic DED. Still, as correctly stated by a recent report and despite the recent recommendations by DEWS [3], “there are no prophylactic medications commonly used to prevent the development of postoperative dry eye” [9]. In our opinion, the main value of the present study is to raise attention to the possible prevention of the iatrogenic DED effects by prophylactic lubricating treatment. To the best of our knowledge, this prevention is a poorly explored topic. In our study, the protective treatment with a lubricating agent started 2 weeks before cataract surgery. This prophylaxis was associated with better homeostasis of the ocular surface on the day of surgery compared to the control group. Such a positive *milieu* may have prevented or reduced the anterior segment inflammation, leading to postoperative protection toward DED signs and symptoms. Of note, the differences between the two groups may have been even higher, considering that group A encompassed the patients (*n* = 3) with AEs. Although these AEs were unrelated to study treatment, they temporarily worsened the postoperative course of these patients.

Our study has some limitations. We did not assess a possible full recovery of ocular surface health due to the lack of a long-term follow-up (2–3 months) after surgery like other trials [4, 5]. However, as well as other authors [5], we have detected the peak of the severity of dry eye conditions in both groups at the 7-day visit post-surgery, and the values of TBUT test and OSDI scores confirm this trend. The lack of a long-term follow-up visit prevented us from evaluating the return to normality of the patients of the control group and estimating a possible shorter recovery time for patients protected with the innovative lubricating agent. Also, we included in the study only subjects with TBUT > 7 s. Future studies may be focused on the performances of the ophthalmic solution VisuEvo^®^ to prevent the worsening of pre-existing DED in different groups of patients, such as those with moderate-to-severe DED or other comorbidities (e.g., high evaporation, blepharitis, glaucoma, corneal neuropathy). Also, it may be interesting to compare VisuEvo^®^ with other tear film substitutes both in iatrogenic and non-iatrogenic DED scenarios.

## Conclusions

The application of a 2-week preoperative treatment with an innovative lipid tear film substitute (VisuEvo^®^) administered twice-daily significantly reduced postoperative DED-related signs and symptoms to almost normal values in elderly subjects undergoing cataract surgery. The clinical value is even more evident by comparing the treated group with subjects receiving standard preparation and postoperative care. The decline of the ocular surface health of the patients in the control group retraced the natural prognosis due to the underestimated postoperative DED.

Further studies will confirm the long-term VisuEvo^®^ efficacy in the complete recovery from postoperative DED damages induced by cataract surgery and explore its potential in the presence of more severe DED.
